# The Role of Training in the Development of Adaptive Mechanisms in Freedivers

**DOI:** 10.2478/v10078-012-0036-2

**Published:** 2012-05-30

**Authors:** Andrzej Ostrowski, Marek Strzała, Arkadiusz Stanula, Mirosław Juszkiewicz, Wanda Pilch, Adam Maszczyk

**Affiliations:** 1 Departament of Theory and Methodology of Water Sports, University School of Physical Education, Cracov, Poland.; 2 Department of Sports Theory, The Jerzy Kukuczka Academy of Physical Education, Katowice, Poland.; 3 Departament of Physiology and Biochemistry, University School of Physical Education, Cracov, Poland.

**Keywords:** freediving, breath-hold, training, adaptive mechanisms

## Abstract

Freediving is a sport in which athletes aim to achieve the longest or the deepest breath-hold dive. Divers are at risk of gradually increasing hypoxia and hypercapnia due to a long time spent underwater and additionally of increasing hyperoxia while depth diving. Exceeding the limits of hypoxia endurance leads to loss of consciousness or even to death whithout immediate first aid. Often enhanced world records indicate the ability to shape specific to the discipline adaptive mechanisms of cardio-pulmonary system which are individually conditioned. During stay underwater heartbeats decelerating called bradycardia, increase in blood pressure, peripheral blood vessels narrowing and blood centralization in freediver’s organism. These mechanisms enhance blood oxygen management as well as transporting it first of all to essential for survival organs, i.e. brain and heart. These mechanisms are supported by spleen and adrenal glands hormonal reactions.

## Introduction

Breath-hold time and water pressure resistance are the two major challenges, which accompany extreme breath-hold diving, called freediving. Freediving is a sport in which athletes aim to achieve the longest or the deepest breath-hold dive. The International Association for the Development Freediving (AIDA International), founded in 1992, organizes competitions, keeps a register of world records set in 8 disciplines and oversees every other activity associated with freediving. The static and the dynamic pool competitions put divers at risk of gradually increasing hypoxia and hypercapnia. Meanwhile, diving in deep and open water makes divers prone to increasing hydrostatic pressure, which may cause higher partial O_2_ pressure and hyperoxia (Muth et al., 2003). While ascending from great depths, a rapid reduction in hydrostatic pressure occurs causes lungs to dilate, additionally with cerebral oxygen desaturation, together with intense hypercapnia (Dujic et al., 2009), may result in a loss of consciousness. Elite freedivers are able to hold their breath for a long time, with oxygen blood saturation decreasing to 50% of the normal saturation level and a simultaneous and significant increase in CO_2_ partial pressure (Ferretti, 2001). This indicates development of adaptive mechanisms that protect divers from anoxia as well as decelerating usage of O_2_ (Joulia et al., 2009). These mechanisms activate themselves during the dive and protect the diver from brain damage or loss of consciousness (Fagius and Sundlof, 1986, Gooden, 1994).

The freediving competition is organized into the following disciplines (World records set, as of 31.12.2011, may be found in the parenthesis and also on the AIDA International (www.aidainternational.org):

- STA (Static Apnea), timed breath-holding in a static position with face submerged in the water (11.35 min – male, 8.23 min – female),
- NLT (No Limits), descending with a sled and ascending with a balloon (214 m – male, 160 m – female),
- DNF (Dynamic Without Fins), distance diving without fins (218 m – male, 160 m – female),
- DYN (Dynamic With Fins), distance diving with fins (273 m – male, 225 m – female),
- VWT (Variable Weight), descending with the help of a ballast weight, enabling faster and easier sinking; and ascending after releasing the ballast and using a rope with simultaneous fin work to pull oneself up (142 m – male, 126 m – female),
- FIM (Free Immersion), depth diving without the use of propulsion equipment, but rather a guiderope during descent and ascent (121 m – male, 88 m – female),
- CWT (Constant Weight), descending and ascending with the help of fins and/or arms without active use of a rope or release of a ballast (124 m – male, 101 m – female),
- CNF (Constant Weight Without Fins), descending and ascending using only muscle strength with constant ballast (101 m – male, 62 m – female).

## The aim of the study

New world records set in breath-hold diving testify to the increasing abilities of the human body, particularly the cardio-respiratory system’s adaptive reactions. The aim of the present study is to assess the role of specialized training in the development of cardio-respiratory adaptive mechanisms that aid volitional respiration and control of breathing. What’s more, this will be a presentation of the human ability to adapt to intense environmental pressure changes experienced while diving. The observed changes in the human organism are applicable to a small group of people that by means of systematic training have acquired a sophisticated ability. The paper provides a review of world literature on related subjects of interest for the authors.

## The functioning of the cardiac system during breath-hold diving

Human functioning is based on constant respiration meant to supply the body with a proper amount of O_2_, which is removed from the organism in form of CO_2_. The breathing rhythm is mainly regulated by peripheral and central chemoreceptors (Sapru, 1996). Despite breath holding, blood circulation ensures that oxygen is still delivered to the tissues and that CO_2_ is expelled from them. With time, gas ratios change; partial O_2_ pressure decreases and partial CO_2_ pressure increases, which leads to a decrease in the amount of O_2_ delivered to tissues, including brain tissue. In the body of an average and regularly breathing man, there is approximately 1500 ml of O_2_ in the tissues, of which about 370 ml resides in the lungs and about 280 ml in blood (Rahn, 1964). In comparison to non-divers, the O_2_ reserve is much higher in the lungs of elite divers – approx. 1000 ml, with a similar amount in hemoglobin (Hb) and small amounts in mioglobin and blood plasma. Thanks to this O_2_ reserve in the lungs, the arterial blood reaching the lung alveola is re-oxygenated, causing arterial blood O_2_ saturation to be still close to 100% in the well-trained athlete, even after a breath-hold for over 3 minutes. Due to O_2_ consumption and a decrease in its partial pressure in the lung alveola, the flowing blood is less and less oxygenated with time. This does not mean that the brain immediately receives less oxygen. Oxygen blood saturation is admittedly lower, yet the blood circulation to the brain is higher, which is caused by the dilation of blood vessels in the brain that occurs with increased CO_2_ concentration. According to Dujic and co-workers (2009) the arterial oxygen saturation (SaO_2_) is reduced with long breath holds, however, the compensatory increase in cerebral blood flow (CBF) could potentially offset the reduced SaO_2_ and maintain the cerebral tissue oxygenation. Research of Palada’s team (Palada et al., 2007) using Near-infrared spectroscopy, which is the method that continuously and noninvasively monitors cerebral oxygenation and cerebral blood volume, as well as the transcranial Doppler technique for observation of CBF shows the cerebral oxygenation is reduced only slightly until the end of the breath hold at the expense of reduced peripheral blood flow and oxygenation.

Chemosensitivity of the peripheral nervous system is the protective mechanism for maintaining cranial perfusion, or the circulation of structural fluid, mostly blood, through tissue or organ. Cranial blood flow (CBF) may potentially compensate for decreased arterial blood O_2_ saturation, enabling the body to maintain proper oxygenation of the brain tissues (Dujic et al., 2010). Intensification of the cranial blood flow is accompanied by the Bohr effect. For a freediver, the Bohr effect signifies that with high CO_2_ concentrations, which occur after a long breath-hold, the blood transfers more O_2_ to tissues; and brain tissue in particular is better supplied with O_2_. 2,3-diphosphoglycerate synthesized in red blood cells as one of the glycolysis product is the factor enhancing the this effect, its level plays an important role in reducing the affinity of hemoglobine to oxygen. Moore and Brewer (1980) say that increased 2,3-DPG levels decrease hemoglobin-oxygen affinity, or shift the oxygen dissociation curve rightward, which can be expected to augment tissue oxygen delivery if arterial oxygen saturation remains high. Hypoxia would appear to be the primary stimulus, but the mechanisms by which hypoxia stimulates an increase in red blood cell 2,3-DPG levels are not clear. It is probably alkalosis, how hypothesize Moore and Brewer (1980), induced by hyperventilation, which in turn stimulates in vivo phosphofructokinase activity or patterns of change in the levels of glycolytic intermediates have been observed during altitude hypoxia – if it is this situation may not occur in freediving.

Over time O_2_ reserves are reduced, causing its decrease in the brain. After exceeding a critical state, a brain susceptible to oxygen deficiency cannot function in an anaerobic way and reacts with unconsciousness. Loss of consciousness is a protective reaction, safeguarding the brain from damage. As a result of unconsciousness, all muscles (except the myocardium) stop functioning, which is meant to save any additional oxygen for brain functioning. This protective mechanism helps to avoid brain damage, even in the case of anoxia or the suspension of the heart beat for more than 4 minutes (Nitka, 2007). After these few minutes, gradual death of the brain tissues and the degradation of the central nervous system occurs. At first the higher functions of the nervous system suffer damage and this is followed by the degeneration of the vegetative functions. Proper safety measures, such as attentiveness to the diver and a quick response to his/her loss of consciousness with resuscitation, may help the diver avoid brain damage due to anoxia during breath-hold diving. It is worth mentioning that the above-mentioned description is based in large part on hypotheses and divers’ observations, as there is no published research, which could adequately and safely measure loss of consciousness in divers.

## Adaptive mechanisms of the cardio-respiratory system occurring in breath-hold diving

The aim of the adaptive mechanisms of the cardio-pulmonary system is to carefully manage oxygen and maintain constant supply to the tissues most susceptible to damage as a result of anoxia, namely the heart and brain tissues. The diving reflex is primarily pulmonary and strictly connected to the reaction of the cardiac system. These changes, noticeable by the freediver, are dependent on the duration of the breath-hold, increased lung pressure after breathing in and facial trigeminal nerve reaction in cold water (Craig and Jr Medd, 1968, Schuitema and Holm, 1988, Sterba and Lundgren, 1988, Schagetay et al., 2007). This complex adaptive mechanism is caused by simultaneous activation of the sympathetic and parasympathetic parts of the nervous system. It consists of bradycardia; peripheral blood vessels narrowing, therefore causing a greater amount of blood to move into the chest; and a hormonal reaction of the adrenal glands, causing increased secretion of catecholamines and the splenetic effect (Fagius and Sundlof, 1986, Foster and Sheel, 2005).

### Bradycardia

Bradycardia is a diver reflex that causes deceleration of the heartbeat and occurs most often in sea mammals (Pelizzari and Tovaglieri, 2009). In spite of weak adaptive mechanisms to water environments in humans (as compared to sea mammals), human deceleration of the heartbeat occurs. Bradycardia is an essential protective reaction of the cardiac system, aimed at economically managing O_2_ levels during breath-hold (Schagatay and Anderson, 1998, Palada et al., 2007). It occurs in cases of general breath-hold as well as during the submersion of the face in water, especially in cold water, and causes increased activity of the face vagus nerve during diving (Schuitema and Holm, 1988, Schagatay et al., 2007, Bosco et al., 2007). Bradycardia, as a response to lowered cardiac minute volume, a significant increase in pressure and the simultaneous contraction of peripheral vessels, develops mainly in the first 30 seconds of apnea (Sterba and Lundgren 1988, Gooden, 1994, Andersson and Schagatay, 1998, Ferrigno et al., 1986 Lindholm and Lundgren, 2009). Butler and Woakes (1987) noted that after 33 seconds of held breath, the heart beat decreases to about 48 beats per minute, and in some cases, even to 20 beats per minute. The moderated and economical use of O_2_ results from a lowered demand on oxygen during slower heartbeats, which causes the decrease of the cardiac minute volume (Bjertnaes et al., 1984). When the heartbeat slows down, increase in myocardium contraction force does not occur, and this does not cause an increase in cardiac minute volume. Decrease in heart rate is thus dependent on the time of breath-hold in water; it is slight after short submersion periods and significant when it exceeds 50 seconds. At the end of the first minute, chest blood vessels are dilated and this correlates with breath-hold duration (Furlan et al., 1993, Saito et al., 2009). But this phenomenon may be caused by higher pressure in the chest as a result of taking a deep breath in before diving, resulting in a reaction from the sympathetic nervous system’s pressure receptors and simultaneously, a lower heart beat. Intensified breathing in, generating an increase in chest pressure, hinders venous blood circulation, thereby decreasing the pulse rate and cardiac minute volume while increasing blood pressure (Ferrigno et al., 1985, Potkin et al., 2007). This effect may be prolonged by glossopharyngeal isufflation, or the practice of holding one’s breath in for a long time, which brings on significantly higher pressure inside the chest (Overgaard et al., 2006). However, blood pressure does not rise remarkably within the first 3 minutes of breath-hold. Increase in blood pressure in consecutive minutes and the reduction of the heart rate coincide with the beginning of the decrease in SaO_2_, which is the result of arterial chemoreceptors reactions significantly rising (Anderson et al., 2009).

Bradycardia in water does not depend on the diving depth, but rather depends on the length of the breath-hold Bosco (2007). During descension, the heart rate decreases until the depth of about 20 m, after which it does not change remarkably. Exceeding 20 m of depth seems to be a critical point in bradycardia reaction, wherein the lung volume decreases due to an increase in water pressure to 1/3 of the maximal volume. Bradycardia occurrence in freedivers cannot be fully explained. It is thought that it may be related to the face receptors by the vagus nerve reaction, contraction of peripheral blood vessels due to sympathetic nervous system reaction, hypoxia caused by apnea or the activity of chemoreceptors in the lungs and large blood vessels (Lin et al., 1983).

### Narrowing of the peripheral blood vessels and blood centralization

The narrowing of the blood vessels, caused by the activation of the sympathetic nervous system during breath-hold diving, causes decreased blood circulation to organs that are more resistant to anoxia, especially the extremities, and to the decrease in PO_2_ in pulmonary alveola and in arterial blood (Fagius and Sundlof, 1986). This mechanism delays development of the heart, lungs and central nervous system (Bosco et al., 2007). With the blood vessels narrowing in the extremities, blood is centralized and results in an increase in blood pressure (Ferretti et al., 1991). Thanks to this effect, the organs that are less vital to the body’s survival consume less oxygen because of their lowered glucose metabolism and reduced lactate production (Behrisch and Elsner, 1984). In states of the maximal narrowing of blood vessels (which occur late in the dive and at deep-levels), the extremities are minimally supplied with blood containing O_2_, and therefore processes that are maintained during this time are those that are based on anaerobic transformation (Craig and Jr Medd, 1968, Sterba and Lundgren, 1988, Pelizzari and Tovaglieri, 2009). Tissues suffering from anoxia may be forced to produce energy using high-energy phosphates, with aerobic metabolism of oxygenated tissues and anaerobic metabolism to produce lactic acid (Ferretti et al., 1991).

The narrowing of the peripheral blood vessels and the simultaneous delivery of large amounts of blood to the chest areas define the phenomenon of lung protection against suppression at large depths, called blood shift. In the first half of the 20^th^ century it was believed that maximal depth for breath-hold diving depended only on vital lung capacity and residual lung capacity. It was also thought that vital lung capacity could not decrease below the residual capacity, because it might lead to lung suppression. But lung suppression in such conditions does not occur. In Craig and Jr Medd (1968), it was stated that physiological compensative mechanisms exist, which protect lungs from suppression. It was also claimed that the chest received more blood due to increased external pressure, and this extra blood was the result of a reduction its amount in peripheral vessels. These reaction mechanisms were confirmed inter alia by Schagatay and Andersson (1998).

Blood shift to lungs occurs after the formation of a pressure gradient between separate body compartments reacting differently to water pressure. As a result of the descent, water pressure causes suppression of the lungs, which are located in the tough and protective rib cage. The chest may sink in to some extent and oppose external water pressure. As consequence, water pressure is balanced by lung air pressure and the resistant force that results from chest elasticity. With an increase in depth, the chest is not able to shrink to such an extent as the compressed lungs. This means that a slight hypotension is constantly present in lungs. In order to compensate for this under pressure, blood from other parts of the body (mainly from the extremities) is pumped to the blood vessels of the chest. A gradual shift of the internal organs from abdomen to chest, as conditioned by diaphragm elasticity, is an additional effect of the blood shift effect. These phenomena cause the chest to be filled with blood and the internal organs, which contrary to lung air are incompressible, protect ribs from fracturing. The blood shift effect and the shift of the intestines towards the compressed lungs increase with depth. Schaffer et al. (1968), on the basis of their research, stated that at the depth of 40 m an additional 850 ml of blood transfers to the lungs. Lung blood vessels, thanks to their elasticity, can expand even to ten-times their original diameter, which is why they can contain even 1,5 l of additional blood, which takes the place of the compressed volume. Data et al. (1983) produced research, which confirmed that an increase in pulmonary artery blood pressure from 16 mm Hg to about 40 mm Hg raises the heart dimensions and causes the diaphragm to ascend with increasing depth. They also indicated that less blood circulates through lower parts of the lungs and more in the middle and upper parts. Bosco et al. (2007) presume that during depth breath-hold diving the “suction” effect and peripheral blood vessel contraction contribute to a huge amount of blood in lungs that may lead to non-uniform blood flow through the ventricles (i.e. the right ventricle pumps more blood than the left one), explaining additional blood transportation to lungs. After the end of the breath-hold dive, chemical reactions of the sympathetic nervous system resume to the initial circulation and respiration regulations, which indicates a lack of permanent changes (Dujic et al., 2008).

## Hormonal reactions

As a result of stress caused by the intense effort that occurs during breath-hold diving, intensive production of organic chemical compounds – catecholamines – occurs in the adrenal glands core and the postganglionic fibres of the sympathetic nervous system. Catecholamines are soluble in water and 50% of these compounds circulate in the blood and are bound to the blood plasma proteins. In the highest levels of concentration, adrenaline, noradrenaline and dopamine are present. Adrenaline acts as a neurotransmitter in the central nervous system and as a hormone regulating blood pressure. Catecholamines induce general reactions in the body, which prepare it for physical effort by increasing blood pressure, accelerating the heart rate and raising the blood glucose level.

As a result of decreased blood perfusion, local ischaemia occurs in the kidneys, causing anoxia, which also stimulates EPO production (Balestra et al., 2006). EPO stimulates proliferation and maturation of bone marrow’s red blood cells. Consequently, the increased number of red blood cells (RBC) enables the body to enhance blood transportation and blood oxygen saturation, which may contribute to enhancing of aerobic endurance, however increase in RBC number depends as well as on soluble in blood plasma transferrin receptor and iron status (Béguin et al., 2003). Moreover during diving, large amounts of erythrocytes are excreted from the spleen, which raises Hct and Hb concentration from 2 to 5% (Jelkmann, 1992).

An hormonal reaction associated with the rise of catecholamines in blood plasma, or the combination of the nervous and hormonal reactions is called the spleen effect (Stewar et al., 2003). The spleen effect is described as a part of the diving reflexes, occurring simultaneously with peripheral blood vessel contraction, blood centralization and bradycardia (Espersen et al., 2002). Additional erythrocytes liberated into the blood stream by the spleen contractions have a supportive effect during multiple breath-hold dives and therefore ”warm-ups”, involving several dozen of apneas that are seconds long, are a vital part of freediving training and a routine pre-start procedure (Schagatay et al., 2001).

The spleen stores blood to a volume that may amount to about 200–300 ml, with 80% of the content consisting of hematocrite (Laub et al., 1993). During the breath-hold, the spleen contracts to the same extent, regardless of whether the diver is above or under water, pumping blood to the cardiac system, and with this, aggregated erythrocytes (Bakovic et al., 2005, Schagatay et al., 2007). The resultant blood oxygen capacity enables an increase in O_2_ concentration by 2.8–9.6% and more intense oxygen transport inter alia to the chest and other organs essential to breath-hold diving (Stewart and McKenzie, 2002, Richardson et al., 2008).

Spleen contraction develops quickly, as it occurs in the first repetition of the breath-hold, and after the next 3 to 4, it reaches its maximum and is very variable (20–46%) and depends on changes in the hypoxia rate (Hurford et al., 1990, Schagatay et al., 2001, 2005, Espersen et al., 2002, Bakovic et al., 2005, Balestra et al., 2006, Prommer et al., 2007). Stronger spleen contractions occur when apnea is connected to face submersion in water but it does not influence the extent of Hb and Hct increase (Espersen et al., 2002). Breath-hold divers in consecutive trials are able to hold their breath for longer periods of time. This may be explained inter alia by the fact that with every apnea the spleen contracts, releasing successive amounts of blood containing red blood cells. An additional number of eythrocytes circulating in blood due to spleen contraction may be equally important to the following: an increase of the O_2_ reserve and available O_2_ supply; an increase in CO_2_ buffering; simultaneous extension of the “easy phase”, and a delay of the unintentional respiratory movements characteristic of the “struggle phase”, or volitional prolongation of the apnea (Schagatay et al., 2005).

The spleen effect may be also caused by other mechanisms and may remain active in short breaks between dives (Hurford et al., 1990, Schagatay et al., 2005). Repeated, multiple breath-hold dives intensify the spleen contraction effect. It shows that hypoxemia enhances spleen and kidney function, increasing Htc and Hb circulating in blood (Schagatay et al., 2007, De Bruijun et al., 2008). For instance, after 3 hours worth of diving, divers experienced a 20% decrease of spleen erythrocytes, causing an increase of Htc by 10% and Hb by 9% in the blood (Hurford et al., 1990).

Three minutes after the end of a dive, the hematological effect of spleen contractions decreases by half and returns to normal dimensions; the initial concentration of Hb and Hct is restored after 10 minutes (Schagatay et al., 2005, Richardson et al., 2008). The additional number of red blood cells leads to blood densification and an increase of blood vessel resistance. Blocking already narrowed blood vessels is a side effect of spleen contraction.

Still researchers do not know much about the real causes of human spleen contraction during breath-hold (Richardson et al., 2009). It is believed that the spleen has adrenoreceptors in its vessel system and adrenaline secretion causes its contraction (Ayers et al., 1972). Another explanation proposes that a decrease in SaO_2_ during breath-hold may be the cause of the mechanism, leading to spleen contraction and the related increase in Hb and Hct (Schagatay et al., 2001, Espersen et al., 2002, Bakovic et al., 2005). Higher blood Hb levels in breath-hold divers may be influenced by the level of the EPO hormone, secreted to the blood stream during intermittent hypoxia, which takes place during freediving. In research conducted by De Bruijn et al. (2008), this higher level was noted; on average it is higher by 24% of the initial value. In this research, the apnea protocol consisted of 15 maximal duration apneas, divided into three series of five apneas. Apneas were spaced out by 2 minutes and the series was interrupted by 10 minutes of rest. In order to reduce pre-apneic alveolar PCO_2_, to prolong apneic duration and to increase arterial oxygen desaturation, subjects hyperventilated for 1 minute prior to each apnea by increasing tidal volume.

## Individual determinants favorable to extreme breath-hold diving

Extreme breath-hold diving depends on many individual determinants. According to one of the world’s most famous free divers, Umberto Pelizzari, extreme results in this sport may be accomplished only by people with a “natural gift” that results from a solid physical and psychological predisposition. Possessing a “natural gift” lays down a favourable foundation for people without health problems, related to cranial sinuses, the cardio-pulmonary system or the respiratory system (Pelizzari and Tovaglieri, 2009).

Vital lung capacity is very important for the practice of freediving, but it is not necessarily essential for it. Good results can be achieved by people with a small vital lung capacity (e.g. Tomoko Yamanouchi of Japan, with a vital capacity of only 2.8 l, achieved in the CWT discipline the depth of 70 m, which is among the best results for females) as well as by people with an extremely large vital lung capacity, which is a characteristic of most breath-hold divers (Seccombe et al., 2006). It is not known whether people owe the good results to genetic predispositions or to the effect of diving training techniques and respiratory exercises, including “lung packing”. It is possible that during training, mobility in the joints and respiratory muscles increases, which causes a rise of the chest dimensions (Tetzlaff et al., 2008). Herbert Nitsch, world record-holder in the NLT discipline, reached vital lung capacity of 14 l after special respiratory training. (www.nautica.pl/freediving/dlaemerytow.htm). It is worth remembering that the average vital lung capacity is 6 l for males and 4 l for females.

Individual determinants are integrally linked to age but contrary to other sport disciplines, it plays a less important role for free divers. Generally speaking, depending on the competition, sportsmen between 20 and 30 years of age succeed the most. Analysis of elite freedivers demonstrated that world records were beaten both by younger athletes, 20–30 years old and by people in their 40’s. Therefore the youth principle is not fully applicable to freediving. It may arise from the fact that long-term training and technical skills in breath-hold diving, develop the body’s adaptive abilities and build experience. The careers of elite freedivers, such as Jacques Mayol and Enzo Maiorca, which have spanned the last 20 to 30 years, may exemplify this fact. These athletes achieved their best results when they were almost 60 years old. J. Mayol set his first world record (60 m in NLT discipline) at the age of 39, and at 56 years old, he dived to the depth of 105 m, which was his best life achievement. Similarly his rival, E. Maiorca, achieved his world record with a dive to the depth of 101 m, which he set at the age of 57. Other examples include Frenchman Andy Le Sauce, who at the age of 53 set a world record in the STA discipline (7.35 min) and in the DYN category (164 m). The American 2003 World record-holder Anabel Briseno, reached 71 m at the age of 53 in the FIM discipline and 6.21 min in the STA discipline. Another example is Bill Graham, a USA record-holder from 2003, who at the age of 64 achieved 6.56 min in the STA discipline. It seems that relaxation and loosening abilities of separate muscle groups, so important in freediving, once mastered and consistently trained, become greater and greater. Moreover, with training, duration tolerance to O_2_ deficiency and CO_2_ excess increases. Similar observations apply to the adaptive abilities of the human organism to high pressures, occurring at great depths. Therefore, even though the overall efficiency of the body may decrease with age, this is compensated by increasingly improved adaptive mechanisms, enabling elderly people to achieve record results as well (www.nautica.pl/freediving/dlaemerytow.htm, Pelizzari and Tovaglieri, 2009).

Young people with relatively little diving experience also can achieve good results in freediving. Frenchman Guillaume Néry at the age of 20 set the 2002 world record in the CWT discipline, reaching the depth of 87 m. The Canadian, Tylor Zetterstroma, after several months of training, achieved almost 8 min in the STA discipline and may be considered another example of this fact.

Present world record-holders were in the mid to elderly age range: Wilian Trubridge (CNF, FIM) – 30 years old; Natalia Molchonova (CNF, CWT, DNF, DYN, STA, FIM) – 46–49 years; Herbert Nitsch (CWT, VWT, NLT) – 37–40 years; Dave Mullins (DNF) – 29 years; Goran Colak (DYN) – 28 years; Stephane Mifsud (STA) – 38 years; Annelie Pompe (VWT) – 29 years; and Tanya Streeter (NLT) – 29 years (www.aidainternational.org). In many instances the clear age difference, which occurs between current world record-holders and those from two decades ago, may indicate huge reserves of the human organism, technical progress, and device and training technique development, which may influence further improvements in the current freediving world records.

The psyche is a very important individual determinant in breath-hold diving, maybe even more important so than physical determinants. The freediver must be mentally resilient to exertion, pain, intensified effort in extreme conditions as well as to the potential threats to health and life. Mental predispositions have particular significance in the “struggle phase”, strongly influencing breath-hold duration (Schagatay et al., 1999). Phobias related to extremely long and deep stays under water, reactions to O_2_ shortage and to CO_2_ excess or to unplanned, potentially dangerous situations under water may generate stress, which is very difficult to overcome or even a disqualifying factor in freediving.

## The role of training in the development of diving reflexes in extreme breath-hold diving

Adaptive reactions of the cardio-respiratory system to hypoxia occur in all people. As a result of specialized training, their efficiency increases and enhances diving abilities (Schagatay et al., 2000). In highly trained freedivers, the sympathetic nervous system activity (MSNA) and blood pressure significantly increase during successively decreased blood oxygen saturation. As a result of training, alveolar partial O_2_ pressure may decrease to 20–40 mm Hg at about 50% of arterial blood O_2_ saturation (Ferretti, 2001, Overgaard et al., 2006, Dujic et al., 2008, Lindholm and Lundgren, 2009). It should also be added that sympathetic nervous system activity in elite freedivers is controlled by different reflex mechanisms than those in people training in other sport disciplines (Heusser, 2009). Breath-hold training causes lower blood acidity, higher tolerance to anoxia, decelerated metabolism and an increase in Hct value, Hb and EPO concentration as well as the mass and volume of the lungs (Schagatay et al., 2000, 2001, 2005, 2007, Bakovic et al., 2005, Prommer et al., 2007, De Bruijn et al., 2008, Richardson et al., 2008).

In highly trained freedivers, who can hold their breath for several minutes as a result of intensified chemical reactions caused by a decrease in SaO_2_, the sympathetic nervous system is activated causing the contraction of peripheral blood vessels (Furlan et al., 1993, Leuenberger et al., 2005, Saito et al., 2009). Intermittent anoxia, which lasts between 20 and 30 minutes, is sufficient enough to intensify sympathetic nervous system activity (MNSA) and increase blood pressure (Leuenberger et al., 2005). Intensified MNSA can be observed even without significant decrease in SaO_2_, which proves that the diving reflex is much more distinct in those people in training, even up to five times more than in people who do not train (Joulia et al., 2003). This helps to maintain increased oxygen supply to the most important organs, especially to the brain and heart, which is interpreted as a mechanism that protects the brain from an insufficient O_2_ concentration in the blood (Joulia et al., 2009).

The increased blood supply to the brain is also caused by the increased density of the capillary vessels, formed as the result of respiratory training (Cavez et al., 2000). Thanks to this fact the breath-hold duration may be extended in spite of the decrease in blood O_2_ saturation (Joulia et al., 2009). According to Dujic et al., (2009) close to the end of the breath hold, an evident drop in cerebral oxygenation occurs, possibly due to the prevailing regional cerebral desaturation, despite the compensatory mechanisms. The cerebral oxygen desaturation, together with intense hypercapnia finally determine the end of the breath hold.

O_2_ consumption is lower during static as opposed to dynamic diving. The unusually long duration of the breath-hold in the STA discipline shows the role of training in achieved results among the elite athletes (Ferretti et al., 1991). Joulia et al. (2003) stated that after three months of regular training, significantly better results in the STA discipline can be achieved, which testifies to the fact that in well-trained divers hypoxemia, hypercapnia and blood acidification symptoms develop more slowly.

Long-term, specialized training also cause an increase in the respiratory system’s functional parameters, which in consequence delays respiratory muscle-fatigue during prolonged breath-hold diving (Nygren-Bonnier et al., 2007). Only highly trained breath-hold divers are able to survive the so-called breaking point, after which the second breath-hold phase, or the “struggle phase”, follows (Andersson and Schagatay, 1998). The moment at which the reflex to increase the frequency of unintentional diaphragm contractions kicks in is dependent on the level of anoxia and blood CO_2_ concentration (Whitelaw et al., 1981). Compression of the chest organs, including the heart, is the consequence of intensified diaphragm contractions, which causes higher pressure, yet is not high enough to limit blood circulation through the valves and aorta. In trained breath-hold divers, short and repeated draughts influence metabolic reactions to occur, which causes a decrease in oxidative stress and lactate acidosis (Craig and Jr Medd, 1968, Qvist et al., 1993). A small amount of lactic acid in the blood in well-trained divers may indicate decreased production or increased catabolism (Joulia et al., 2002). In chronic hypoxia, lactate release is decreased but this mechanism cannot be fully explained. It may result from decreased production and faster lactic acid removal from oxygen deficient muscles (Bender et al., 1989). Some research indicates that exposure to chronic hypoxia boosts metabolism and activates use of free fatty acids released during physical effort, which spares the muscle glycogen reserves and thus decreases lactic acid production (Young et al., 1982). Occurring after apnea, stalling the degradation of muscle cells due to blood lactate acidosis is most likely one of the adaptive mechanisms to prolonged breath-hold duration (Joulia et al., 2003). Thus, the experience of a decrease in blood lactate concentration during breath-hold training may mean that a reduction in muscle production or an increase in oxygenation in other tissues is occurring. It means that a decrease in oxygen consumption by the muscles and organs is followed by the decrease of aerobic metabolism. Research presented by Joulia et al. (2002) indicates lower glutatione with thiobarbituric acid (TBARS) levels and ascorbic acid concentration after the physical activity. It suggests that repeated hypoxia periods, occurring in elite breath-hold divers, causes a more benign process of oxidative stress, while a stronger intensification of the cellular membranes’ protective mechanism from lipide peroxydation (Joulia et al., 2003). Lower oxygen flow to the tissues increases reactive oxygen, producing more free radicals and causing cellular membrane lipids peroxidation (Dhaliwal et al., 1991). Temporary anoxia and local inflammatory reactions to microinjuries, in working muscles hampering proper cellular metabolism, are responsible for oxidative stress during physical effort (Sjödin et al., 1990, Steinberg et al., 2002, Bosco et al., 2007). Apnea, responsible for the contraction of peripheral blood vessels, causes a decrease in O_2_ levels in the muscle, which explains the significant decrease in oxidative stress in divers (Joulia et al., 2002). The abovementioned authors observed also that during relaxation after apnea training, oxidative stress is lowered and indicated by the decrease in the TBARS concentration (Joulia et al., 2003).

It seems that acute anoxia does not intensify the membranes’ lipid peroxidation but even may reduce it. Sjödin et al., (1990) proposed the hypothesis that a decrease in O_2_ supply to muscles reduces production of free oxygen radicals.

Intensive breath-hold diving training (e.g. 5–6 h a day, 6 days a week for 6 months) sufficiently stimulates EPO increase, which causes higher red blood cells concentration and greater abilities to hold one’s breath for longer periods of time (Lemaître, 2009). Delay in arterial blood O_2_ desaturation development, preventing the most important organs from functioning during multiple breath-hold dives, leads to the conclusion that these actions may enhance diving reflexes, by enhancing O_2_ management processes (Vasar and Kingisepp, 1980).

## Summary

Everybody who can swim may practice breath-hold diving, even if frequently it is a short descent, lasting maximally a few dozen seconds and to a few dozen meters. Freediving is an extreme form of breath-hold diving, and it is a sports competition in which athletes compete for the longest stay under water in a static position or for covering the longest distance in a horizontal or vertical direction. Divers associated with AIDA International compete in eight disciplines, achieving unimaginable results for an average man. Since 1992, frequently beaten world records are an example of this. We advise carefulness for teenagers until age of 15–17 due to biological development of their lowered resistance of nervous system for enhanced exposition of apnea.

Breath-hold diving results are limited mainly by the hypoxia, hypercapnia and additionally (at great depths) hyperoxia that increase over the depth of dive. The results of hypoxia and hypercapnia depend on protective adaptive mechanisms, to a certain extent. Adaptive mechanisms are activated as a result of chemical blood changes causing activation of the sympathetic and parasympathetic nervous system that in turn starts up the diving reflex, consisting of bradycardia; peripheral blood vessel contraction; blood centralization; a hormonal reaction in the adrenal glands; intensified catecholamine secretion; and the spleen effect. The aim is first and foremost to protect sensitive and essential for survival organs, such as the brain and heart, from harmful effects of progressive hypoxemia and hypercapnia.

Research on the differences in the extent of these adaptive mechanisms shows the influence of many factors shaping them. These include: training level, psycho-physical predispositions as well as diving type. Many researchers (Steward, McKenzie 2002, Bakovic et al., 2003, Palada et al., 2007, Heusser et al., 2009 and others) pay attention to the level of training leveling athletes. They posit that breath-hold divers, after consistent training, adapt to extreme hypoxia and hypercapnia by developing better vital lung capacity, the ability to properly ventilate before submersion, maintaining apnea in the “struggle phase” after successfully enduring the “easy phase” and breaking point. The strongest diving reflexes and hormonal reactions occur during the “struggle phase”. The important role that training plays in breath-hold diving is confirmed by the lower frequency of blood acidosis and the decreased level of oxidative stress related to the production of free oxygen radicals. This is yet another piece of evidence that training in freediving is vital, since both lactate acidosis and oxidative stress hinder cell and organ functioning (Joulia et al., 2002).

It is said that the diver’s age is related to his/her training level, skills and experience. However, there is no straightforward confirmation of this assumption because both young people (20–30 years old) and much older (in their 50’s and 60’s) achieve great freediving results. It may be supposed that in younger athletes record-setting results are mainly related to psycho-physical factors, while in older divers experience and technical skills play a large role.

The frequency and extent of adaptive mechanisms depends on the diving discipline. They are less intense in static disciplines (Schagatay and Andersson, 1998) and more intense during dynamic diving (Butler and Woakes, 1987).

It should be emphasized that breath-hold diving and the recurring hypoxia and hypercapnia related to it, do not negatively influence the sympathetic nervous system, blood circulation and respiration, if the person does not suffer from additional risk factors, such as hypertension, glucose intolerance or hyperlipidemia. Although extreme divers are not permanently exposed to underwater conditions, it can be totally excluded that intensified reactions of sympathetic nervous system during long-term breath hold can have negative effect on the cardiovascular system (Breskovic et al., 2010).

It seems that the human can dive to even deeper depths and for longer amounts of time. The limits of human capabilities are difficult to define. Eric Fattah, one of the former world record holders in the CWT discipline, postulated in the web service *Deeper Blue* that the use of yoga techniques in the STA discipline may cause some to set world records numbering several hours. Moreover, in the NTL discipline, it might be possible to descend to over 1000 m, however due to the risk of nitric anesthesia, diving should be performed while breathing out. Lastly the CNF discipline might achieve the depth of 150 m. Eric Fattah claims that results depend not only on the diver but also on the equipment used, mainly the fins. In 10 or 20 years, they might be produced out of materials that will enable more efficient diving. It is unknown if Eric Fattah’s daring forecasts will be confirmed in the future, but one thing is certain, the sport of freediving will be advanced incredibly in the years to come (www.nautica.pl/freediving/dlaemerytow.htm).
